# PD239 Assisted Daily Living: Stakeholder Involvement In Identifying Innovation Gaps, User Needs, And Potential Solutions

**DOI:** 10.1017/S0266462324004562

**Published:** 2025-01-07

**Authors:** Aalya Al-Assaf, Eugenie Evelynne Johnson, Louise Tanner, Emily Hunter, Catherine Richmond, Madeleine Still, Sean Gill, Sara Jackson, Bethan Harris, Oluwatomi Arisa, Barbara Hanratty, Jennifer Liddle, Dawn Craig, Fiona Pearson

## Abstract

**Introduction:**

Technology is central in supporting older people with their daily tasks and independence at home. This project aimed to identify technologies that can be built into residential environments (e.g., appliances, fixtures, or fittings) to support older people in activities of daily living (ADL) through a horizon scan (HS) informed by public insights on unmet needs and priorities.

**Methods:**

A survey of members of the public was conducted to prioritize outcomes included within an evidence and gap map (EGM) framework. The EGM aimed to illustrate the current landscape of technologies supporting ADL in residential settings (e.g., care homes) and innovation gaps. The EGM results were shared with end users in a workshop discussion on the current range of technologies aimed at supporting ADL in residential settings. This was facilitated using vignettes to elicit views on unmet needs and priorities for technology development. The workshop informed the scope of the HS to identify and prioritize emerging technologies that could address unmet needs.

**Results:**

This project successfully embedded public involvement throughout to identify innovation gaps in technologies supporting ADL, unmet needs among end users, and potential solutions to these needs. The HS identified 190 technologies that were ready to market. All the technologies had potential to address identified unmet needs and could be built into the residential environment to support older people with ADL and to improve their quality of life, independence, and safety at home. Horizon scanning research can meaningfully involve stakeholders and take direction from their insights to enable voices less often heard to drive innovation in areas where it is needed.

**Conclusions:**

Involving stakeholders in research using evidence synthesis and qualitative methods helps to gain a better understanding of gaps in innovation, the related unmet needs, and the technologies that might address these needs. Public involvement in the survey and workshop influenced the conduct and interpretation of the EGM, the scope of the HS, and the interpretation of the findings.

